# Attachment of Single-Stranded DNA to Certain SERS-Active Gold and Silver Substrates: Selected Practical Tips

**DOI:** 10.3390/molecules26144246

**Published:** 2021-07-13

**Authors:** Edyta Pyrak, Kacper Jędrzejewski, Aleksandra Szaniawska, Andrzej Kudelski

**Affiliations:** 1 Faculty of Chemistry, University of Warsaw, 1 Pasteur St., 02-093 Warsaw, Poland; epyrak@chem.uw.edu.pl (E.P.); k.jedrzejewsk6@student.uw.edu.pl (K.J.); aleksandra.szaniawska@uw.edu.pl (A.S.); 2 Nencki Institute of Experimental Biology of Polish Academy of Sciences, 3 Pasteur St., 02-093 Warsaw, Poland

**Keywords:** surface enhanced Raman spectroscopy, SERS, DNA, thiolated DNA, gold nanoparticles, silver electrodes

## Abstract

Layers formed from single-stranded DNA on nanostructured plasmonic metals can be applied as “working elements” in surface–enhanced Raman scattering (SERS) sensors used to sensitively and accurately identify specific DNA fragments in various biological samples (for example, in samples of blood). Therefore, the proper formation of the desired DNA layers on SERS substrates is of great practical importance, and many research groups are working to improve the process in forming such structures. In this work, we propose two modifications of a standard method used for depositing DNA with an attached linking thiol moiety on certain SERS-active structures; the modifications yield DNA layers that generate a stronger SERS signal. We propose: (i) freezing the sample when forming DNA layers on the nanoparticles, and (ii) when forming DNA layers on SERS-active macroscopic silver substrates, using *ω*-substituted alkanethiols with very short alkane chains (such as cysteamine or mercaptopropionic acid) to backfill the empty spaces on the metal surface unoccupied by DNA. When 6-mercapto-1-hexanol is used to fill the unoccupied places on a silver surface (as in experiments on standard gold substrates), a quick detachment of chemisorbed DNA from the silver surface is observed. Whereas, using *ω*-substituted alkanethiols with a shorter alkane chain makes it possible to easily form mixed DNA/backfilling thiol monolayers. Probably, the significantly lower desorption rate of the thiolated DNA induced by alkanethiols with shorter chains is due to the lower stabilization energy in monolayers formed from such compounds.

## 1. Introduction

In recent years, there has been much research activity in developing new techniques enabling genetic disorders to be diagnosed through the identification of DNA chains having a given sequence in various biological samples (for example, in blood). Various techniques can be used for such identification [1,2,3,4,5,6]. One group of very promising, ultrasensitive methods that can be used to sensitively and accurately identify specific DNA fragments consists of methods that utilize surface-enhanced Raman scattering (SERS) [7,8,9,10,11,12,13,14,15,16,17,18,19]. In SERS spectroscopy, the Raman signal generated by molecules adsorbed on certain nanostructured plasmonic materials is increased by many orders of magnitude. The SERS phenomenon is explained as a result of a synergistic cooperation of two mechanisms: a local enhancement of the intensity of the electric field, which is a consequence of the excitation of the localized surface plasmons, and an increase in the efficiency of the generation of Raman scattering due to chemical interactions with the SERS substrate, in an effect similar to standard resonance Raman scattering [20,21]. Important characteristic features of the enhancement of the SERS spectrum are a sharp decrease with increasing distance from the SERS substrate, and in the case of an agglomeration or aggregation of plasmonic SERS-active nanostructures, a very large increase in the SERS activity of the material (a very large field enhancement is observed in the narrow slits between the plasmonic nanograins, known as “hot spots”, and the formation of such structures significantly increases the SERS activity of the material) [20,21]. SERS is one of the most sensitive analytical tools, and in some cases, it is possible to observe a quality SERS spectrum of even a single molecule [22,23].

There are many different approaches used for detecting specific DNA chains based on measurements of SERS signals, including the following:

(i) A moiety with an exceptionally large cross-section for Raman scattering (known as a Raman reporter) is connected to the target DNA, and after hybridization with the capture DNA, this target DNA with a Raman reporter is immobilized on the SERS substrate and a strong SERS signal is recorded [7,8].

(ii) A capture DNA hairpin chain is covalently bonded via one end to the SERS substrate, and a Raman reporter is attached to the other end of the hairpin chain, which is close to the SERS substrate; hybridization between the target DNA and the capture DNA forming the hairpin disrupts the stem-loop configuration and causes a decrease in the SERS signal of the Raman reporter [9,10,11].

(iii) Capture DNA is adsorbed on a SERS substrate, and this then hybridizes with the target DNA; subsequently, a hybridization with a second “auxiliary DNA chain” labelled with a Raman reporter is carried out, resulting in a sandwich structure giving a strong SERS signal [12,13,14,15].

(iv) Two different capture DNA chains are immobilized on two various sols of SERS-active nanoparticles; when the target DNA is present in the sample, an aggregation of the plasmonic nanoparticles is induced by the target DNA, which leads to a significant increase in the intensity of the measured SERS spectrum [16,17,24].

(v) A modification of the method described in point (iv), where instead of one modified nanoparticle, a macroscopic plasmonic substrate [25] or magnetic nanoparticles [26] are used.

(vi) The application of head-flocked gold nanopillar SERS substrates modified with a capture nucleic acid; in the presence of the target nucleic acid, the substrates self-assemble and form SERS hot spots [27].

(vii) Capture DNA is immobilized on a SERS-active surface via thiol linking moiety; hybridization with the target DNA induces a change in the conformation of the alkanethiolate linking moiety and in its SERS spectrum [18].

In all these SERS DNA sensors, the active part of the device contains a layer of “capture” DNA attached via a thiol linking moiety to a SERS-active substrate, with additional alkanethiols that fill the “empty” spaces on the metal surface between the DNA chains. Moreover, this kind of “capture” DNA layer on a plasmonic metal is also used in sensors that utilize a surface plasmon resonance signal as an analytical response [28]. For these reasons, the formation of DNA layers on SERS-active plasmonic substrates is being studied and developed by many groups around the world. In this contribution, we propose two modifications of this process leading to layers that yield a stronger SERS signal of DNA: (1) freezing the sample during the adsorption of DNA with an attached thiol linking moiety on plasmonic nanoparticles [29,30,31], and (2) in the case of the formation of DNA layers on SERS-active macroscopic silver substrates (silver substrates usually generate a stronger SERS enhancement factor than the gold substrates commonly used [32]), using a compound other than 6-mercapto-1-hexanol (normally used on standard gold substrates) to fill the places on the metal surface unoccupied by DNA [18,33,34].

## 2. Results and Discussion

As mentioned in the introduction, we decided to analyze how certain modifications of the method for depositing thiolated DNA on plasmonic nanoparticles might affect the intensity and repeatability of the SERS spectra recorded, and how the chemisorption of various alkanethiols used to fill those places on the metal surface unoccupied by DNA could affect the structure of the obtained layer.

### 2.1. Modification of the Adsorption Procedure

**Figure 1:**
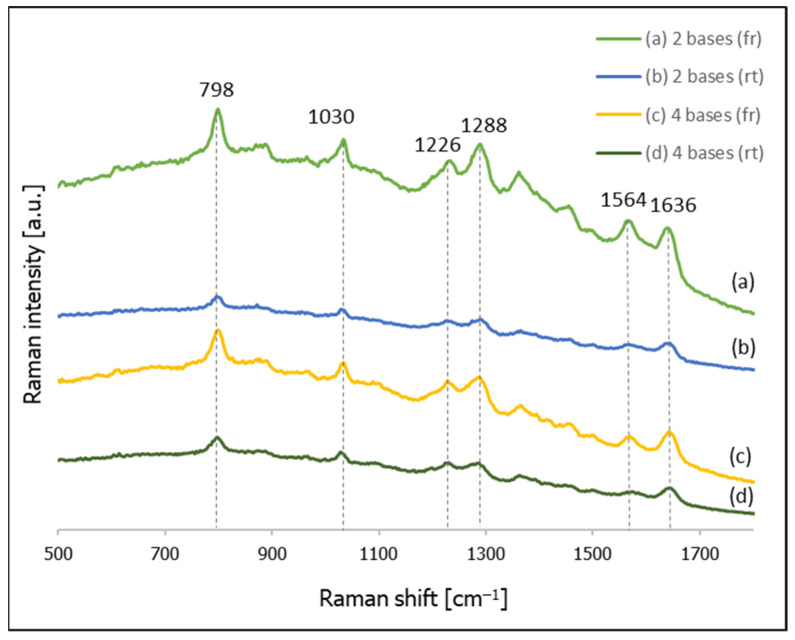
SERS spectra of thiolated DNA on gold nanoparticles: (**a**) CT–(CH_2_)_6_–SH (with freezing the sample), (**b**) CT–(CH_2_)_6_–SH (prepared at room temperature), (**c**) CTCT–(CH_2_)_6_–SH (with freezing the sample), (**d**) CTCT–(CH_2_)_6_–SH (prepared at room temperature).

As mentioned in the introduction, it is well-known that thiols chemisorb on a surface of gold and silver, forming strong sulphur-metal chemical bonds [35,36,37,38,39]. Therefore, regarding DNA chains attached to the metal surface, a linkage moiety containing a –S–H group is often attached to the DNA chain, and such a modified compound is then chemisorbed on the surface of the metal (usually gold). The standard procedure for adsorbing thiolated DNA on SERS-active nanostructured gold involves mixing a diluted solution of thiolated DNA with a sol of Au nanoparticles at room temperature, and waiting a few hours to allow the reaction (between the –S–H groups and the metallic nanoparticles) to proceed and the thiolated DNA to self-assemble. We observed that a small modification of the process of self-assembly of thiolated DNA may significantly increase the intensity of the SERS spectra recorded. Figure 1 shows sample SERS spectra of layers formed from CT–(CH_2_)_6_–SH and CTCT–(CH_2_)_6_–SH on gold nanoparticles. In all cases, the solution of thiolated DNA and a sol of gold nanoparticles was mixed and kept overnight; either at room temperature or in a freezer (in the case of the frozen samples, they were thawed before the SERS measurements, which were carried out at room temperature).

As can be seen from Figure 1, the SERS spectra of the samples that were frozen before the SERS measurements are about 4 times more intense than the spectra of the samples prepared at room temperature. Analogous experiments have also been carried out for the following thiolated DNA: C–(CH_2_)_6_–SH, CTC–(CH_2_)_6_–SH, CTCTG–(CH_2_)_6_–SH, CTCTGT–(CH_2_)_6_–SH, CTCTGTA–(CH_2_)_6_–SH, CTCTGTAG–(CH_2_)_6_–SH, CTCTGTAGC–(CH_2_)_6_–SH, CTCTGTAGCT–(CH_2_)_6_–SH, CTCTGTAGCTA–(CH_2_)_6_–SH and CTCTGTAGCTAG–(CH_2_)_6_–SH (sequences are given from 5′ to 3′). To quantitatively estimate the freezing-induced increase in the intensity of the recorded SERS spectra, in all the obtained spectra we determined the integral intensity of the band at ca. 800 cm^−1^, which is due to the ring breathing vibration of the pyrimidine ring of cytosine [40] (we chose the strongest cytosine band since, in all cases, the DNA chains contained cytosine). We found that in the experiments carried out for all the above-mentioned thiolated DNA, the SERS spectra measured for the frozen samples were stronger. The average increase determined from the spectra recorded for all the DNA chains was 5.5, which means that freezing the sample when preparing DNA layers on gold nanoparticles for SERS measurements may result in samples generating SERS spectra that are significantly stronger than those from samples prepared at room temperature. We also found that the reproducibility of the SERS spectra of the samples prepared using a freezing cycle is not worse than that of samples prepared at room temperature.

**Figure 2:**
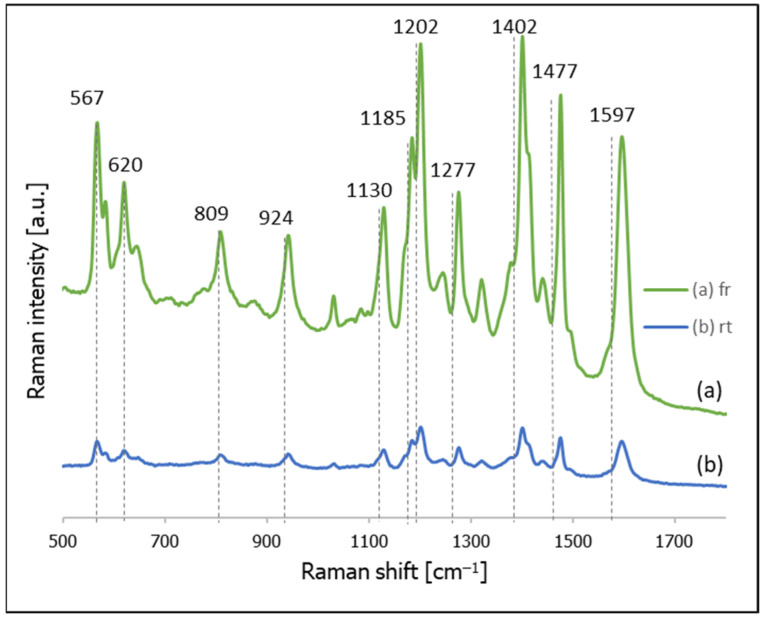
SERS spectra of thiolated DNA modified with Cy3 at the 5′ end adsorbed on gold nanoparticles: (**a**) using a freezing step, and (**b**) room temperature incubation.

Since DNA generates a relatively weak Raman signal, in many cases of SERS sensors utilizing DNA, what is known as a Raman reporter moiety (a chromophore having a very large cross-section in Raman scattering) is attached to the DNA chain. Figure 2 compares the SERS spectra of thiolated DNA to which a Raman reporter moiety (cyanine Cy3) was introduced for samples self-assembled at room temperature or using a freezing step. As can be seen in this Figure, in the case of thiolated DNA with attached cyanine Cy3, the sample prepared using freezing generated a significantly (approx. 8 times) more intense Raman signal than the sample prepared at room temperature.

**Figure 3:**
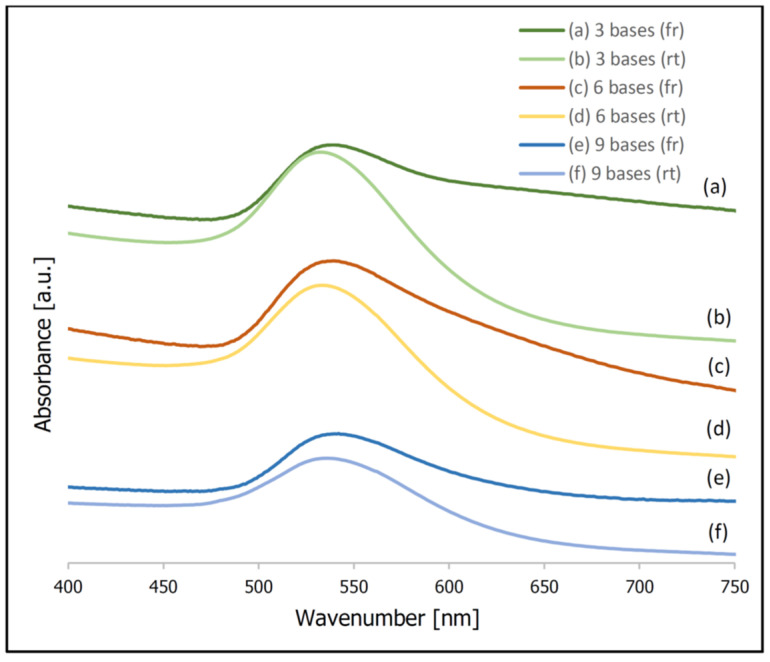
Normalized UV-Vis spectra of gold nanoparticles covered with thiolated DNA. The adsorption of DNA was carried out using a freezing step (spectra (**a**,**c**,**e**)) and at room temperature (spectra (**b**,**d**,**f**)). Experiments for: (**a**,**b**) 3 bases; (**c**,**d**) 6 bases; (**e**,**f**) 9 bases.

**Figure 4:**
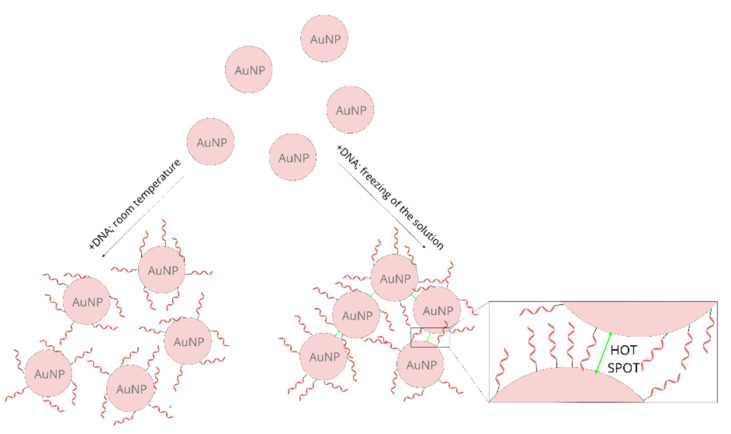
A scheme showing the difference between the samples prepared at room temperature and those prepared using a freezing step.

In addition to the described above difference in the SERS activity of the samples prepared using a freezing step and incubated at room temperature, a significant difference in the extinction spectra of the samples prepared using these two methods was observed. Figure 3 shows normalized UV-Vis extinction spectra of gold nanoparticles covered with thiolated DNA (this Figure presents data obtained in experiments using DNA chains composed of three, six and nine bases). It can be seen that the position of the plasmonic band for the samples prepared at room temperature is at 533–534 nm. Whereas, for the samples prepared using a freezing step the same band appears at 540–542 nm. Moreover, for the samples prepared using a freezing step, a significant increase in the extinction of the radiation in the red part of the visible spectrum is observed. Both these spectral changes are characteristic of an aggregation of plasmonic nanoparticles [41,42]. It can be deduced that, for the samples prepared using a freezing step, the degree of aggregation is significantly larger. Since the aggregation of plasmonic nanoparticles significantly increases their SERS activity (especially large SERS enhancements are observed for molecules in the narrow slits between the plasmonic nanoparticles, known as “hot spots” [20,21]), a certain agglomeration of the gold nanoparticles after the freezing step is probably also a cause of the larger SERS activity of such samples. During freezing, water crystals form, which cause a concentration of the solution (meaning a local concentration of the gold nanoparticles and DNA). This facilitates the chemisorption of the thiolated DNA on the metal nanoparticles, and at the same time facilitates the agglomeration of the nanoparticles (Figure 4 shows a schematic difference between the samples prepared at room temperature and prepared using a freezing step).

**Figure 5:**
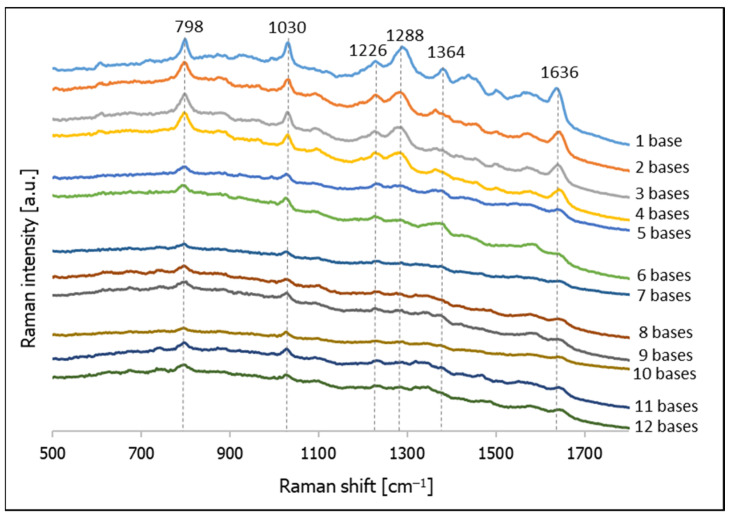
SERS spectra of thiolated DNA having various chain length deposited on gold nanoparticles using a freezing step.

The majority of SERS signals are generated in SERS hot spots [43,44], which, for plasmonic nanoparticles, usually appear in the narrow slits between the nanoparticles. Therefore, changing the thickness of the layer of DNA deposited should affect the width of the slits formed, and should therefore have an impact on the intensity of the SERS signal. Figure 5 shows SERS spectra of gold nanoparticles with the following thiolated DNA deposited using a freezing step: C–(CH_2_)_6_–SH, CT–(CH_2_)_6_–SH, CTC–(CH_2_)_6_–SH, CTCT–(CH_2_)_6_–SH, CTCTG–(CH_2_)_6_–SH, CTCTGT–(CH_2_)_6_–SH, CTCTGTA–(CH_2_)_6_–SH, CTCTGTAG–(CH_2_)_6_–SH, CTCTGTAGC–(CH_2_)_6_–SH, CTCTGTAGCT–(CH_2_)_6_–SH, CTCTGTAGCTA–(CH_2_)_6_–SH, CTCTGTAGCTAG–(CH_2_)_6_–SH. As can be seen from this Figure, there is a significant decrease in the intensity of the measured SERS spectra when the chain of deposited thiolated DNA contains more than 4 bases (although a larger number of nucleic bases are present around the gold nanoparticles surrounded by DNA with a longer chain, a less intense SERS signal is generated). One may assume that the wider slits created by the gold nanoparticles surrounded by a thicker layer of DNA are not as efficient in enhancing the SERS spectra.

We also analyzed the SERS spectra obtained for the investigated thiolated DNA. When the DNA contains only cytosine and thymine bases (from 1 to 4 bases), the most intense bands in the spectra are due to the following vibrations; at 798 cm^−1^ due to the cytosine ring breathing vibration; at 1030 cm^−1^ due to the N–sugar stretching vibration; at 1288 cm^−1^ due to the CN stretching vibration; at 1500 cm^−1^ due to the NH_2_ deformation vibration; at 1564 cm^−1^ due to the pyridine ring stretching; and at 1636 cm^−1^ due to C=O stretching vibrations (see also Table 1) [40,45,46]. In the SERS spectra of DNA with strains containing five and six bases, we did not observe any new Raman bands assigned to guanine, although the intensity of the measured spectra clearly decreased. Some of the less intense bands visible in the first four spectra were no longer observable. New weak bands assigned to the vibrations of adenine clearly appeared in the spectrum of the DNA containing seven bases: the ring breathing vibration band at 742 cm^−1^ and the band due to the C=N stretching vibration in the imidazole ring at 1466 cm^−1^ [40,45,46]. As can be seen from Figure 4, the SERS spectra of the DNA that have a longer chain are generally similar. The assignments of the most intense Raman bands appearing in the SERS spectra of various thiolated DNA adsorbed on gold nanoparticles are listed in Table 1.

We also investigated whether freezing the solution during DNA deposition had any impact on the intensity of the SERS signal recorded on the macroscopic nanostructured plasmonic substrates (for these experiments we chose an electrochemically nanostructured silver electrode). In such a case, we did not observe any significant differences between the SERS spectra of the silver electrode stored at room temperature and the silver electrode after freezing. This observation also supports the hypothesis that an increase in the intensity of the SERS spectrum after freezing is caused by the agglomeration of plasmonic nanoparticles (such an agglomeration is not possible in the case of macroscopic SERS substrates).

### 2.2. Influence of Chemisorption of Various Alkanethiols Used for Preventing Contact between the DNA Backbones and the Metal Surface

**Figure 6:**
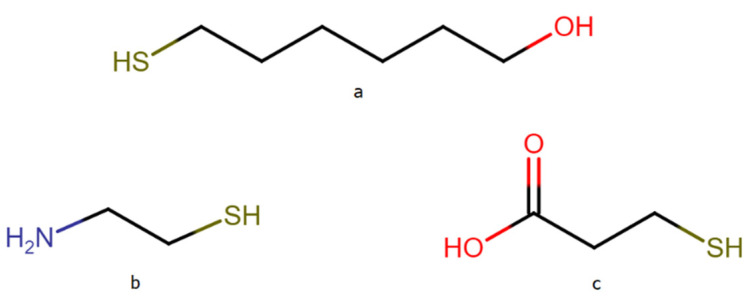
Structure of the alkanethiols: (**a**) 6-mercapto-1-hexanol (MCH), (**b**) cysteamine (CYS), (**c**) 3-mercaptopropionic acid (MPA).

DNA attached to a metal surface via a thiol moiety tends to interact with the metal surface not only through the thiol groups, but also through the nitrogen bases. This effect hinders the operation of DNA sensors, because it disturbs the hybridization process with other DNA strains. To eliminate direct interactions of the DNA nitrogen bases with the metal surface, certain compounds can be used to “backfill the empty spaces” on the metal surface between the adsorbed DNA chains (a surface density of the DNA chains that is too high is also undesirable because it disturbs the hybridization process with the target DNA strains). In the case of the formation of DNA layers on gold (gold is the most widely used substrate for the formation of DNA layers for various sensors), the empty spaces are usually backfilled using chemisorbed 6-mercapto-1-hexanol (MCH) [18,33]. Yet, because silver nanostructures usually generate a stronger SERS enhancement factor than gold nanostructures [32], which makes silver substrates very promising for SERS sensors, we decided to verify whether, in the case of experiments conducted on nanostructured silver, MCH could be also used as an efficient compound for filling the spaces on the metal surface unoccupied by DNA. The chemical structures of the alkanethiols used in this work are presented in Figure 6.

**Figure 7:**
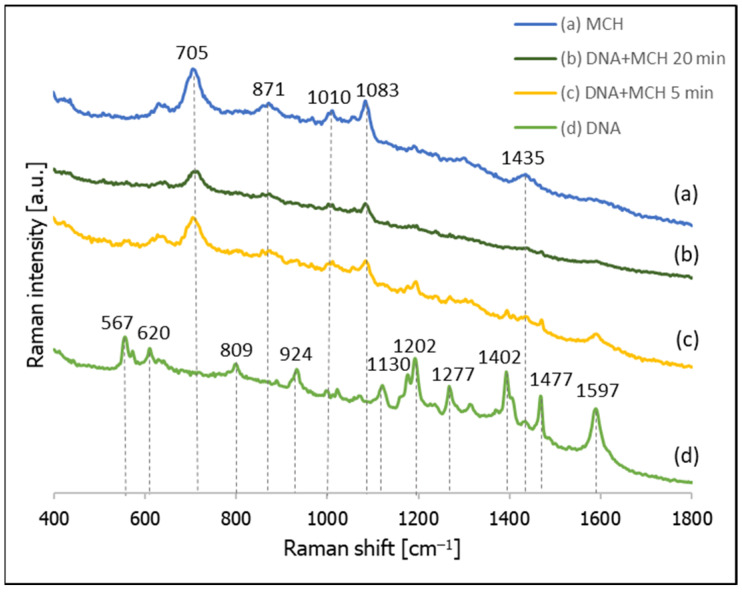
SERS spectra of layers formed on a nanostructured silver electrode from: (**a**) a 10 mM MCH solution, (**b**) thiolated DNA labelled with Cy3 and immersed for 20 min in a 10 mM MCH solution, (**c**) thiolated DNA labelled with Cy3 and immersed for 5 min in a 10 mM MCH solution, and (**d**) thiolated DNA labelled with Cy3.

Cy3 labelled thiolated DNA was adsorbed on a nanostructured silver electrode by overnight incubation. Next, the SERS spectrum of the layer of DNA formed on the silver was measured, and the substrate was then immersed in a 10 mM MCH solution (pH 4.5) for 5 min and 20 min. After soaking in the MCH solution, the SERS spectrum of the sample was measured again. In a separate experiment, a nanostructured silver substrate was immersed for 5 min in a 10 mM solution of MCH, and the SERS spectrum of the modified substrate was measured. All the obtained spectra are presented in Figure 7. As can be seen from this Figure, although Cy3 has a very large cross-section for Raman scattering and yields very strong SERS spectra, even when slightly separated from the metal surface, when a silver substrate covered with thiolated DNA with an attached Cy3 moiety was soaked for 5 min in a 10 mM MCH solution, the resulting spectrum contained MCH bands only at 705, 871, 1010, 1083 and 1435 cm^−1^, which is practically identical to the SERS spectrum of MCH adsorbed on a silver surface. Moreover, even decreasing the concentration of MCH by one order of magnitude (to 1 mM) did not prevent the chemisorbed DNA from being removed from the silver surface—See Figure S1 (Supplementary Materials). This suggests that MCH exchanges thiolated DNA on the silver surface, and therefore may be inappropriate for preparing DNA monolayers on silver.

Due to the immersion of a thiolated DNA layer on silver in a 10 mM (or even in a 1 mM) MCH solution induces an efficient desorption of DNA from the metal surface, which means that this compound seems to be inappropriate for backfilling the empty spaces on the metal surface. On this basis, we decided to check whether a similar quick desorption of DNA would be also observed when soaking a layer formed by the chemisorption of thiolated DNA in solutions of two other simple *ω*-substituted alkanethiols: 3-mercaptopropionic acid and cysteamine. We chose substituted alkanethiols with very short chains in order to decrease the very large stabilization effect that takes place during the self-assembly of alkanethiols with long alkane chains, which is due to the alkane chains forming crystal-like structures.

**Figure 8:**
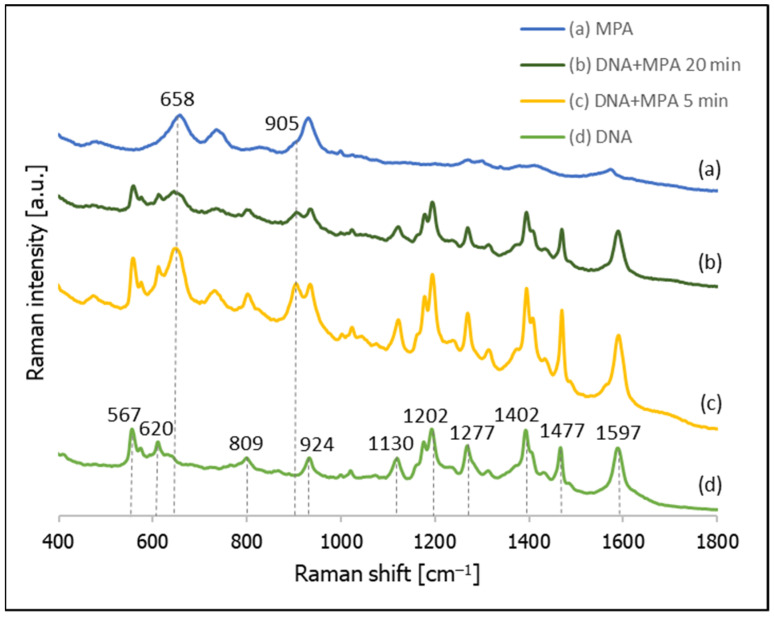
SERS spectra of layers formed on a nanostructured silver surface from: (**a**) a 10 mM MPA solution, (**b**) thiolated DNA labelled with Cy3 and immersed for 20 min in a 10 mM MPA solution, (**c**) thiolated DNA labelled with Cy3 and immersed for 5 min in a 10 mM solution of MPA, and (**d**) thiolated DNA labelled with Cy3.

Figure 8 shows the SERS spectra recorded from a nanostructured silver surface modified by chemisorbed thiolated DNA with an attached Cy3 moiety, 3-mercaptopropionic acid (MPA), and the temporal evolution of the SERS spectrum of a layer formed from thiolated DNA with an attached Cy3 moiety after immersion in a 10 mM solution of MPA (pH = 2.8). As can be seen from this Figure, after 5 min of incubating the DNA-modified silver electrode in the MPA solution, we can observe both the previously observed bands due to the DNA and the appearance of new bands that are due to the MPA (especially the band at 658 cm^−1^ due to the S–C stretching vibrations of the gauche conformer of the S–C–C chain [37,38,39,47] and the band at 905 cm^−1^ due to the vibration of the non-dissociated carboxylic group vibrations [47]). In addition, 20 min of soaking in the solution of MPA did not induce a significant desorption of the DNA (DNA bands can even be seen for the DNA samples soaked in MPA for 60 min, although in these cases the bands due to MPA definitely dominate the spectrum).

**Figure 9:**
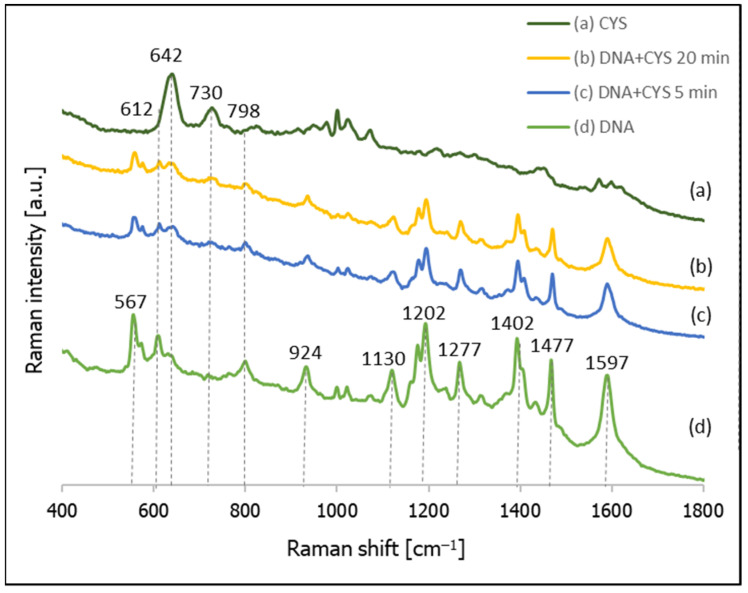
SERS spectra of layers formed on a nanostructured silver surface from: (**a**) a 10 mM cysteamine solution, (**b**) thiolated DNA labelled with Cy3 and immersed for 20 min in a 10 mM cysteamine solution, (**c**) thiolated DNA labelled with Cy3 and immersed for 5 min in a 10 mM cysteamine solution, and (**d**) thiolated DNA labelled with Cy3.

**Figure 10:**
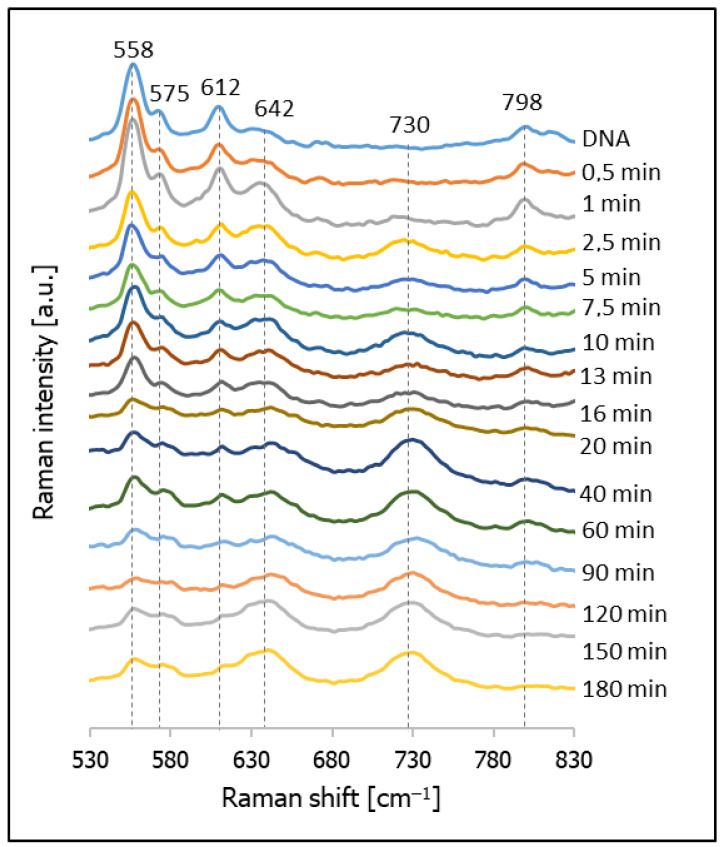
SERS spectra of layers formed on a SERS-active silver surface previously modified with thiolated DNA labelled with Cy3 (the spectrum marked DNA shows the spectrum of the electrode covered with DNA before immersion in a CYS solution) after immersion for various times in a 10 mM CYS solution.

As mentioned above, the second compound tested in this contribution as a possible compound for backfilling the empty spaces in DNA layers was cysteamine (CYS). Figure 9 shows the temporal evolution of the SERS spectrum of a nanostructured silver surface modified by chemisorbed thiolated DNA with an attached Cy3 moiety after immersion in a 10 mM CYS solution, and the SERS spectrum of a monolayer formed only from CYS. As can be seen from this Figure, the Raman bands due to the vibrations of DNA are clearly visible in all the measured spectra, which means that, in this case, the DNA was not removed very quickly from the silver surface, as in the case of experiments with MCH. Moreover, Raman bands characteristic of adsorbed molecules of CYS are clearly visible, even in the spectrum accumulated after just 5 min of immersing the DNA layer on silver in a 10 mM CYS solution (pH = 5). In the next step, we decided to conduct a more careful investigation of the adsorption of CYS molecules between the DNA chains, and to measure the temporal evolution of the SERS spectrum of this system during soaking in a 10 mM CYS solution lasting from 0.5 to 180 min (see Figure 10). We found that, even after 3 h of soaking in the CYS solution, the bands due to the DNA were still clearly visible, which means that the cysteamine did not induce a disconnection of the thiolated DNA from the metal surface. A comparison of the intensity ratios of the bands at 612 cm^−1^ (DNA) and 642 cm^−1^ (CYS) and the bands at 798 cm^−1^ (DNA) to 730 cm^−1^ (CYS) shows that these ratios stabilized relatively quickly (in less than 20 min). This means that semi-stable Ag/thiolated-DNA/CYS systems (semi-stable because, of course, a very long immersion in the CYS solution would certainly lead to the desorption of the DNA molecules) are formed relatively quickly, and CYS is a very promising compound for backfilling the “empty spaces” on a silver surface between adsorbed thiolated DNA chains.

We also carried out analogous experiments concerning the application of these back-fillers using plasmonic nanoparticles. Unfortunately, the adsorption of back-fillers on nanoparticles with, or even without, adsorbed DNA significantly decreased the stability of the sols, and so we were unable to obtain reliable results in these cases.

## 3. Materials and Methods

Analytical grade NaH_2_PO_4_, Na_2_HPO_4_, and KCl were purchased from Chempur, Piekary Slaskie, Poland. Analytical grade cysteamine hydrochloride, 97% 6-mercapto-1-hexanol, 99% 3-mercaptopropionic acid, chloroauric acid and sodium citrate tribasic dehydrate were purchased from Sigma, Poznan, Poland. The platinum plate was purchased from Mennica Polska, Warsaw, Poland. The water used in all the experiments was purified in a Millipore Milli-Q system (Merck Millipore, Burlington, MA, USA).

All the single-stranded DNA used in this work was purified using HPLC (high-performance liquid chromatography) and was purchased from Genomed, Warsaw, Poland. Nucleotides having the following sequences were used (all the listed sequences are from 5′ to 3′): C–(CH_2_)_6_–SH (1), CT–(CH_2_)_6_–SH (2), CTC–(CH_2_)_6_–SH (3), CTCT–(CH_2_)_6_–SH (4), CTCTG–(CH_2_)_6_–SH (5), CTCTGT–(CH_2_)_6_–SH (6), CTCTGTA–(CH_2_)_6_–SH (7), CTCTGTAG–(CH_2_)_6_–SH (8), CTCTGTAGC–(CH_2_)_6_–SH (9), CTCTGTAGCT–(CH_2_)_6_–SH (10), CTCTGTAGCTA–(CH_2_)_6_–SH (11), CTCTGTAGCTAG–(CH_2_)_6_–SH (12), and Cy3–CGAGATTTCTCTGTAGCTA–(CH_2_)_3_–SH. All the nucleotides were dissolved in a phosphate-buffered saline with a Na^+^ concentration of 25 mM. The concentration of nucleotides in the final solution was 50 mM.

Spherical gold nanoparticles with a diameter of about 35 nm were prepared using the following method: 99 mL of water was mixed with 1 mL of a 1% aqueous solution of HAuCl_4_ and heated to boiling under reflux. Then, 1 mL of a 1% aqueous solution of sodium citrate was added. The reaction mixture was stirred and heated to boiling point under reflux for 1 h and then cooled to room temperature. Transmission Electron Microscopy (TEM) images of the resulting gold nanoparticles are presented in the Supplementary Materials—see Figure S2. For the preparation of the DNA probes on gold nanoparticles, 5 μL of the solution of nucleotides was added to 100 μL of the solution of Au nanoparticles. The obtained mixture was stored overnight, either at room temperature or in a freezer. Next, the frozen probes were thawed, and all the samples were centrifuged for 10 min at 2000 *g*. The supernatant was removed, and 3 μL of the concentrated solution was deposited on Au-covered glass slides and the SERS spectra of the liquid probes recorded.

**Figure 11:**
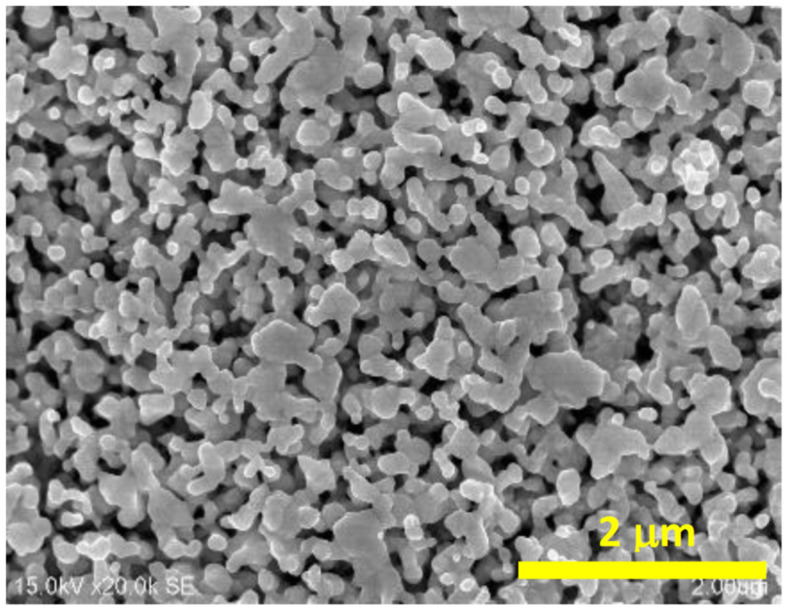
Scanning Electron Microscopy image of the nanostructured silver electrode.

The silver electrodes were roughened by three successive positive-negative polarization cycles in a 0.1 M KCl aqueous solution, from 0.3 V to −0.3 V at a scan rate of 5 mV s^−1^, and then the electrode was kept for 5 min at −0.4 V. All potentials are given versus the 0.1 M KCl AgCl/Ag electrode. A Scanning Electron Microscopy (SEM) image of the nanostructured silver electrode obtained is presented in Figure 11. For the preparation of the DNA films on the silver electrodes, the roughened electrode was immersed in a 10 µM solution of the DNA for 24 h, and then the modified electrode was rinsed with water and the SERS spectra of the modified Ag surface were recorded.

Raman spectra were accumulated using a Thermo Fisher DXR Raman spectrometer (Waltham, MA, USA). The excitation radiation (*λ* = 633 nm) was generated by an He–Ne laser, and the power at the sample was 2 mW. Spectra were recorded using an air objective (10x) with a numerical aperture of 0.25—The diameter of the laser spot at the sample was 2.5 micrometers. For the experiments on the gold nanoparticles, at least five measurements were performed for each sample, and all the SERS spectra presented in this work are the average of at least five spectra collected at different points of the sample.

The absorption spectra were recorded with a UV–Vis-NIR Perkin Elmer spectrophotometer (model Lambda 650, Waltham, MA, USA) in a range of 400–800 nm with a resolution of 2 nm. To place a sample, quartz cells were used.

The electrochemical preparation of the silver electrodes was performed in a conventional three-electrode cell using a PGSTAT204 potentiostat/galvanostat from Metrohm Autolab BV, operated using NOVA 1.10 software.

## 4. Conclusions

For both the labeled and label-free DNA chains, the SERS spectra obtained for the samples prepared using a freezing step were more intense than the spectra of the samples prepared by the standard method of incubation at room temperature. This was probably due to the formation of aggregates of plasmonic nanoparticles, which led to the creation of a larger number of SERS hot spots. This means that using a freezing step for preparing DNA probes on gold nanoparticles seems to be a promising way of preparing samples for SERS measurements.

**Figure 12:**
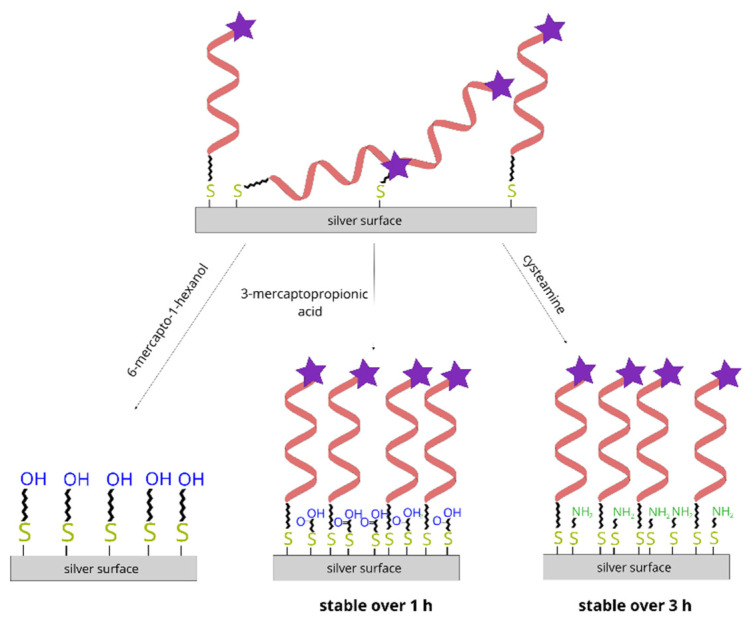
Diagram showing the different impact of soaking the DNA layer on silver in solutions of 6-mercapto-1-hexanol, 3-mercaptopropionic acid and cysteamine.

6-mercapto-1-hexanol, commonly used to fill in the “unoccupied” places on a gold surface covered with a thiolated DNA monolayer, does not seem to be an effective compound for backfilling the empty spaces between DNA chains on SERS-active silver surfaces, because it induces a quick detachment of chemisorbed DNA from the silver surface. We propose that, for DNA layers on silver, *ω*-substituted alkanethiols with significantly shorter alkane chains may be more suitable (for example, 3-mercaptopropionic acid and cysteamine)—See also Figure 12. The lower desorption rate of thiolated DNA induced by these compounds is likely due to the lower stabilization energy in the monolayers formed from such compounds.

## Figures and Tables

**Table 1:** Assignments of the most intense Raman bands appearing in the SERS spectra of various thiolated DNA adsorbed on gold nanoparticles deposited using a freezing step. The numbers from 1 to 12 refer to the DNA chain length.

DNA Composed of Following Number of Bases:												Assignments [40,45,46]
1	2	3	4	5	6	7	8	9	10	11	12	
1636	1642	1640	1642	1638	1640	1643	1641	1638	1638	1641	1644	C=O
1564	1571	1571	1576		1580		1578	1580	1571		1576	ring str (C)
						1548				1550		ring str (A)
1500	1501	1498	1500									NH_2_ def
						1466				1467		C=N str (Im)
1457	1455	1452										C=N str (Py)
1380	1363	1366	1363	1364	1364	1378		1368		1376		C-N str (Py)
							1319			1319	1319	C-N str (Im)
1288	1281	1285	1279	1280	1284		1276	1288	1282		1286	C-N str
1228	1228	1226	1226	1230	1226	1231		1227	1231	1230	1226	C-N str
1030	1030	1030	1030	1027	1026	1028	1029	1027	1026	1027	1026	N-sugar
798	797	798	796	795	792	796	796	801	796	796	798	ring br (C)
						734	743	742	738	742	735	ring br (A)
							675	673		678	676	ring br (G)

## Data Availability

Original spectra are available from E.P.

## Supplementary Materials

The following are available online, Figure S1: SERS spectra of layers formed on a nanostructured silver electrode from DNA labelled with Cy3 and MCH. Figure S2: Transmission Electron Microscopy images of used gold nanoparticles.

## Sample Availability

Samples of SERS substrates are available from the authors.
