# Supplementary material for: Attachment of Single-Stranded DNA to Certain SERS-Active Gold and Silver Substrates: Selected Practical Tips
Source: Molecules. 2021 Jul 13;26(14):4246. doi: 10.3390/molecules26144246 (PMC8305401; doi:10.3390/molecules26144246)
Supplement: Supplementary file 1 [file molecules-26-04246-s001.zip › molecules-1259442-supplementary.pdf]

Supplementary materials

Attachment of single-stranded DNA to certain SERS-active gold and silver substrates: selected practical tips

Edyta Pyrak[1,2], Kacper Jędrzejewski[1], Aleksandra Szaniawska[1], and Andrzej Kudelski [1,*]

[1] Faculty of Chemistry, University of Warsaw, 1 Pasteur St., 02-093 Warsaw, Poland;
[2] Nencki Institute of Experimental Biology of Polish Academy of Sciences, 3 Pasteur St., 02-093 Warsaw, Poland

[*] Correspondence: akudel@chem.uw.edu.pl

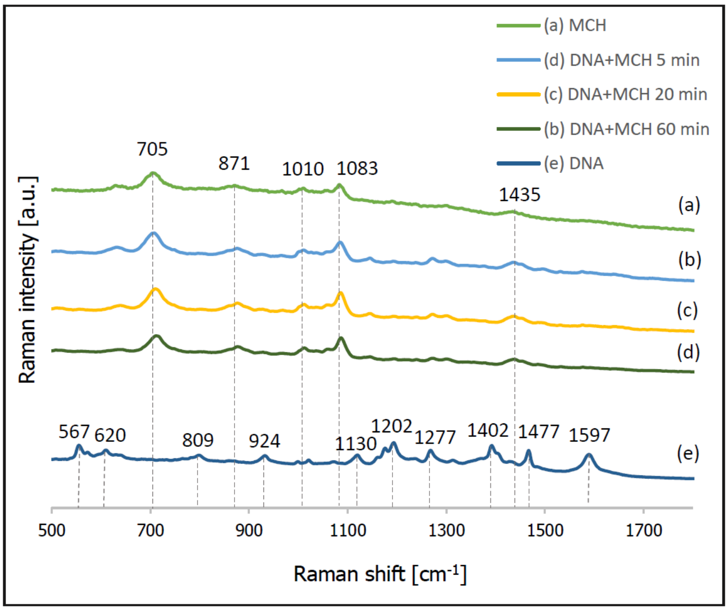

Figure S1. SERS spectra of layers formed on a nanostructured silver electrode from: a) a 1 mM MCH solution, b) thiolated DNA labelled with Cy3 and immersed for 60 min in a 1 mM MCH solution, c) thiolated DNA labelled with Cy3 and immersed for 20 min in a 1 mM MCH solution, d) thiolated DNA labelled with Cy3 and immersed for 5 min in a 1 mM MCH solution, and e) thiolated DNA labelled with Cy3.

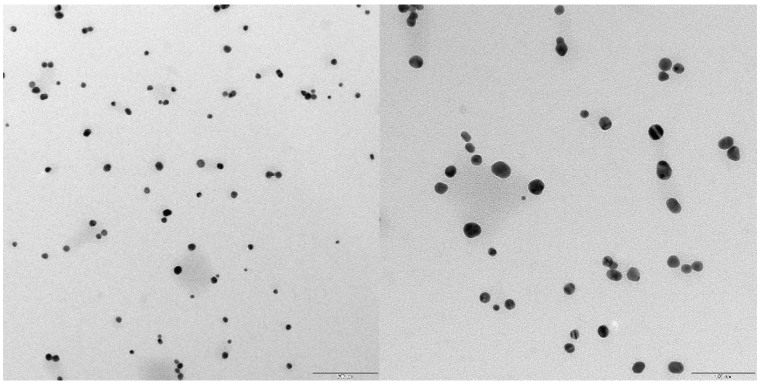

Figure S2. Transmission Electron Microscopy (TEM) images of the gold nanoparticles.
